# Araufuranone: A New Phytotoxic Tetrasubstituted Dihydrofuro[3,2-b]furan-2(5*H*)-One Isolated from *Ascochyta araujiae*

**DOI:** 10.3390/biom12091274

**Published:** 2022-09-10

**Authors:** Marco Masi, Angela Boari, Francisco Sautua, Marcelo Anibal Carmona, Maurizio Vurro, Antonio Evidente

**Affiliations:** 1 Department of Chemical Sciences, University of Naples Federico II, Complesso Universitario Monte Sant’Angelo, Via Cintia 4, 80126 Napoli, Italy; marco.masi@unina.it; 2 Institute of Sciences of Food Production, National Research Council, Via Amendola, 122/O, 70126 Bari, Italy; angela.boari@ispa.cnr.it (A.B.); maurizio.vurro@ispa.cnr.it (M.V.); 3 Phytopathology, University of Buenos Aires, Buenos Aires C1053, Argentina; sautua@agro.uba.ar (F.S.); carmonam@agro.uba.ar (M.A.C.)

**Keywords:** *Araujia hortorum*, *Ascochyta araujiae*, weed biocontrol, fungal metabolites, araufuranone

## Abstract

*Araujia hortorum* is a perennial vining plant species native to South America. It was introduced into many countries for ornamental and medicinal purposes as well as for its edible fruits, but it has become highly invasive, generating severe environmental problems. Biological control using bioherbicides and natural compounds is an interesting control option. The pathogenic fungus *Ascochyta araujiae*, isolated from infected leaves of *A. hortorum*, could be considered as a potential biocontrol agent. Its ability to produce bioactive metabolites was studied. The organic extract of the fungal culture filtrates showed interesting phytotoxic activities consisting of clearly visible necrotic symptoms (0.5–1 cm in diameter) in the punctured leaves. Thus, it was purified; this afforded three main metabolites. These were chemically and biologically characterised: one proved to be a new pentasubstituted dihydrofuro[3,2-b]furan-2(5*H*)-one, named araufuranone (**1**). The others were the already known fungal metabolites neovasinin and 2,4-dihydroxy-6-hydoxymethylbenzaldehyde (**2** and **3**). The structure of araufuranone was determined using spectroscopic methods (essentially 1D and 2D ^1^H and ^13^C NMR and HR ESIMS spectra); its relative configuration was assigned by a NOESY spectrum. To the best of our knowledge, araufuranone is the first example of a naturally occurring compound showing that carbon skeleton. Assayed by a puncture, araufuranone proved to be weakly active on the leaves of *Diplotaxis* sp. and *Sonchus* sp.; the other two metabolites were even less toxic. Tested on cress, compounds **2** and **3** were able to partially inhibit rootlet elongation whereas araufuranone was almost inactive.

## 1. Introduction

*Araujia hortorum* E. Fourn. (fam. Apocynaceae) (often referred to as *Araujia sericifera* Brot.) is a perennial fast-growing vining plant species native to South America. It was introduced into many countries (e.g., Europe, South Africa, and New Zealand among others) for ornamental and medicinal purposes as well as for its edible fruits, but it has become highly invasive, generating serious environmental problems mainly in natural habitats [1,2,3,4,5,6,7].

The search for biological control agents led to proposal of the rust fungus *Puccinia araujiae* Lev. in New Zealand [8,9,10]. More recently, *Ascohyta araujiae* Speg. was identified in Argentina as the causal agent of necrotic leaf spots and defoliation of *A. hortorum* [11]. It was evaluated, together with the fungus *Septoria araujiae*, as a possible biocontrol agent of the weed, alone or in combined treatments [12]. Considering that *A. araujiae* is a necrotrophic pathogen, and after observing a few of the caused symptoms (i.e., necrotic spots and chlorosis), the possible production and release of bioactive phytotoxic metabolites by the fungus during the infection process was hypothesised. Therefore, the fungus was grown in vitro and its culture filtrates, having promising phytotoxic activities, were extracted and purified.

This manuscript reports the isolation and chemical and biological characterisation of the bioactive metabolites produced by *A. araujiae* grown in vitro.

## 2. Materials and Methods

### 2.1. General Experimental Procedures

Optical rotations were measured using a Jasco P-1010 digital polarimeter (Tokyo, Japan). The IR spectra were recorded as a glassy film on a Perkin-Elmer (Waltham, MA, USA) Spectrum One Fourier transform infrared (FTIR) spectrometer. The UV spectra were recorded on a JASCO V-530 (Tokyo, Japan) spectrophotometer in a CH_3_CN solution. The ^1^H and ^13^C NMR spectra were recorded in CD_3_OD at 400 or 500 and 100 or 125 MHz on Bruker (Karlsruhe, Germany) and Varian (Palo Alto, CA, USA) instruments using the same solvent as the internal standard. The carbon multiplicity was determined by a DEPT spectrum [13]. The COSY-45, HSQC, HMBC, and NOESY spectra were recorded using Bruker and Varian microprograms [13]. The HRESI and ESI mass spectra and liquid chromatography (LC)/MS analyses were performed using the LC/MS TOF Agilent 6230B HPLC 1260 Infinity system (Milan, Italy). The HPLC separations were performed with a Phenomenex LUNA (C18 (2) for 5 μ at 150 × 4.6 mm. Analytical and preparative TLCs were carried out on silica gel (Kieselgel 60, F_254_; 0.25 and 0.5 mm, respectively) or on reverse phase (Whatman, KC18 F_254_; 0.20 mm) plates (Merck, Darmstadt, Germany). The spots were visualised by exposure to UV radiation or by spraying first with 10% H_2_SO_4_ in MeOH and then with 5% phosphomolybdic acid in EtOH, followed by heating at 110 °C for 10 min. Column chromatography was performed using silica gel (Kieselgel 60, 0.063–0.200 mm) (Merck).

### 2.2. Fungal Isolates and Culture Conditions

The strain of *Ascochyta araujiae* used in this study was isolated from the necrotic leaf spots of *Araujia hortorum* sampled at San Andrés de Giles, Buenos Aires, Argentina. The fungus pathogen was identified based on previously described morphological characteristics such as the colony growing in potato glucose agar, pycnidia, and conidia [12,14] Afterwards, the internal transcribed spacer (ITS) region and the large subunit (LSU) of the nuclear ribosomal RNA were partially sequenced. The ITS1-5.8S-ITS2 region (ITS) of the nuclear ribosomal DNA operon was amplified and sequenced with the V9G [15] and ITS4 [16] primer pair and the primer combination of LR0R [17] and LR7 [18] was used for the LSU amplification and sequencing. For future reference, the nucleotide sequence data were deposited in the NCBI GenBank database under the accession numbers OP237030 (ITS) and OP237029 (LSU). To our knowledge, these are the first records of gene sequences available for *A. araujiae*. The strain was stored and grown in the laboratories of the Institute of Sciences of Food Production, CNR, Bari, Italy. For the production of bioactive metabolites, the fungus was preliminarily grown in different growth conditions. Among those tested, the best proved to be a defined mineral medium, M-1-D [19]. The fungus from actively growing colonies was transferred into Erlenmeyer flasks (1 L) containing 300 mL of the cited medium and grown in shaken conditions for 20 days at 25 °C in the dark. The mycelium was removed by filtration and the liquid cultures were lyophilised prior to the extraction procedure.

### 2.3. Extraction and Purification of A. araujiae Metabolites

The lyophilised culture filtrate (6.4 L) of *A. araujiae* was dissolved in bi-distilled H_2_O at 1/10 of the initial volume (pH 7.1) and extracted with EtOAc (3 × 500 mL). The organic extracts were combined, dried (Na_2_SO_4_), and evaporated under a reduced pressure, giving a residue of 830.5 mg. This was purified by silica gel column chromatography and eluted with chloroform-*iso*-propanol (9:1), yielding twelve homogeneous fractions (F1–F12). The residue of F5 (27.1 mg) was further purified by TLC eluted with chloroform-*iso*-propanol (9:1), yielding an amorphous solid (**3**, 8.5 mg). The residue of F7 (43.7 mg) was further purified by TLC on a reverse phase eluted with acetonitrile–water (1:1), yielding two amorphous solids (**1**, 4.2 mg and **2**, 5.3 mg).

*Araufuranone* (**1**): amorphous solid; IR ν_max_ 3502, 1675, and 1595 cm^−1^; UV λ_max_ (log ε) 220 (3.2) nm; ^1^H and ^13^C NMR spectra, see Table 1. HRESIMS: (+), *m*/*z*: 305.1370 [M + Na]^+^ (calcd. for C_15_H_22_NaO_5_ 305.1365).

*Neovasinin* (**2**): amorphous solid; [α]^25^_D_—99.8 (*c* 0.16, MeOH); IR ν_max_ 3565, 1670, 1591, and 1239 cm^−1^; UV λ_max_ (log ε) UV λ_max_ 211 (4.5), 240 (3.6), and 290 (4.0) nm; ^1^H NMR, δ: 5.17 (1H, dd, *J* = 9.3 and 1.2 Hz, H-9), 4.70 (1H, d, *J* = 15.1 Hz, H14A), 4.40 (1H, d, *J* = 15.1 Hz, H-14B), 3.78 (1H, s, H-7), 2.41 (1H, m, H-10), 1.91 (3H, s, Me-13), 1.79 (3H, d, *J =* 1.2 Hz, Me-16), 1.38 (3H, s, Me-15), 1.29 (2H, m, H_2_-11), 0.97 (3H, d, *J* = 6.3 Hz, Me-17), and 0.90 (3H, t, *J* = 7.3 Ha, Me-12); ^13^C NMR, δ: 164.3 (s, C.1), 161.8 (s, C-3), 155.7 (s, C-5), 136.2 (d, C-9), 131.1 (s, C-8), 107.6 (s, C-4), 98.8 (s, C-2), 87.7 (d, C-7), 68.2 (s, C-6), 61.7 (t, C-14), 33.2 (d, C-10), 29.9 (t, C-11), 20.6 (q, C-15), 20.5 (q, C-17), 13.3 (q, C-16), 11.9 (q, C-12), and 9.1 (q, C-13); ESIMS (+), *m*/*z*: 347 [M + K]^+^, 331 [M + Na]^+^, and 309 [M + H]^+^; ESIMS (−), *m*/*z*: 307 [M − H]^−^.

*2,4-Dihydroxy-6-hydroxymethylbenzaldehyde* (**3**): amorphous solid; IR ν_max_ 3419, 3240, 1637, 1588, 1491, and 1462 cm^−1^; UV λ_max_ (log ε) 240 (4.0) and 304 (4.2) nm; ^1^H NMR, (CDCl_3_) δ: 9.39 (s, HCO), 6.94 (d, *J* = 2.0 Hz, H-5), 6.20 (d, *J* = 2.0 Hz, H-3), and 4.78 (s, *CH*_2_OH); ESIMS: (+), *m*/*z*: 191 [M + Na]^+^.

### 2.4. Phytotoxic Assays

#### 2.4.1. Bioassay on *Phelipanche ramosa* Seeds

For the bioassay on the parasitic weed *Phelipanche ramosa* (L.) Pomel, a protocol for seed conditioning and germination was used [20]. The filters were cut into smaller square pieces, each containing around 100 conditioned seeds, and placed in Petri dishes. A solution (0.5 mL) with the metabolites (0.1 mg metabolite/mL solution) and the synthetic stimulant (GR24) were added over the filters. The Petri dishes were kept in the dark at 25 °C to allow germination. After five days, the number of germinated seeds was determined in comparison with the control.

#### 2.4.2. Bioassay on Punctured Leaves

The leaves of six weed species (namely, *Calamintha* sp., *Cyperus* sp., *Convolvulus arvensis* L., *Diplotaxis* sp., *Heliotropium europeum* L., and *Sonchus* sp.) were harvested from plants growing in naturally infested fields in the countryside of Bari (Southern Italy). Uniform leaves were chosen and then immediately placed in polycarbonate boxes on suitable trays. The leaf surface was injured using an insulin syringe needle, thus provoking small and superficial wounds immediately before the droplet application. Each leaf received 30 μL of the solution containing 0.8 mg metabolite/mL H_2_O. Three replicates were prepared for each treatment and for each plant species. The boxes were kept at ±25 °C under continuous light. The eventual appearance of the symptoms of phytotoxicity was observed up to 5 days.

#### 2.4.3. Bioassay on Garden Cress (*Lepidium sativus* L.) Seeds

Garden cress (*Lepidium sativus* L. fam. Brassicaceae) seeds were purchased from Emanuele Larosa Seeds (Andria, Italy). This species was chosen because of its high germinability and uniform seedling growth. Briefly, the metabolites were dissolved in methanol and then diluted in methanol–water (2:98, *v*/*v*) to obtain a final concentration of 0.2 mg/L. The *L. sativus* seeds were sanitised with a 1% sodium hypochlorite solution (*v*/*v*) for 10 min and soaked with distilled water. Batches of ten seedlings (one batch for each treatment) were transferred to the filter paper in small Petri dishes (6 cm in diameter). Each filter was moistened with 1 mL of the solution. Each treatment was replicated three times. The plates were placed in a growth chamber at 25 °C with a photoperiod of 12 h light/12 h dark for 3 days. Methanol (2% in water) was added as a control treatment. After 3 days, the germination percentage and the rootlet lengths were measured in comparison with the untreated control.

## 3. Results and Discussion

**Figure 1:**
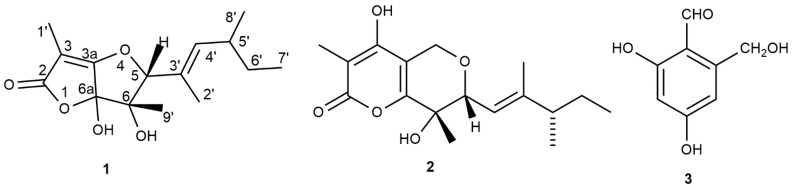
Structures of araufuranone (**1**), neovasinin (**2**), and 2,4-dihydroxy-6-hydroxymethylbenzaldehyde (**3**).

In a first screening comparing the different cultural conditions (shaken vs. still; liquid vs. solid), the shaken liquid culture was identified as the best for the production of bioactive filtrates. The purification process of the phytotoxins was bioguided using the three different bioassays to test the phytotoxicity, as detailed described in the “Section 2”. It began with the assay by a leaf puncture of six weed species (namely, *Calamintha* sp., *Cyperus* sp., *Convolvulus arvensis* L., *Diplotaxis* sp., *Heliotropium europeum* L., and *Sonchus* sp.) of the fungal culture filtrates and the organic extract. Both showed an interesting phytotoxicity consisting of clearly visible necrotic symptoms (0.5–1 cm in diameter); the corresponding aqueous phase showed no activity, demonstrating that the extraction process was exhaustive. The organic extract was then purified by column chromatography and all the fractions collected were assayed for phytotoxicity. Among them, only the active fractions were further purified by TLC to obtain the three pure metabolites, whose chemical and phytotoxic activity are described in the present manuscript. Two of them were known fungal metabolites and were identified as neovasinin and 2,4-dihydroxy-6-hydoxymethylbenzaldehyde, as reported below. The third was a new pentasubstituted dihydrofuro[3,2-b]furan-2(5*H*)-one, and was named araufuranone (**1**, Figure 1).

Araufuranone (**1**) had a molecular formula of C_15_H_22_O_5_, as deduced from its HR ESIMS spectrum, and was consistent with five hydrogen deficiencies. The preliminary investigation of its ^1^H and ^13^C NMR spectra showed the presence of signals typical of carbonyl, olefinic, hydroxylated methine, aliphatic methylene, and vinyl and aliphatic methyl groups [21,22]. These structural features agreed with the bands due to the hydroxy, carbonyl, and olefinic groups observed in the IR spectrum [23] and the absorption maxima due to the extended conjugated groups measured in the UV spectrum [21].

In particular, the ^1^H and COSY spectra (Table 1 and Figures S1 and S2) [13] showed the presence of a double doublet (*J* = 9.4 and 1.2 Hz) at δ 5.22 of an olefinic proton (H-4′) belonging to a trisubstituted double bond, which, coupled with the adjacent methine proton (H-5′) observed as a multiplet at δ 2.43.

The latter proton (H-5′), also coupled with the protons of the adjacent methyl (H_3_-8′) and methylene (H_2_-6′) groups, which resonated as a doublet (*J* = 6.9 Hz) and a multiplet at δ 0.98 and 1.29, respectively. The multiplet of H_2_-6′ also coupled with the methyl group (H_3_-7′) appeared as a triplet (*J* = 7.3 Hz) at δ 0.91, and represents the terminal carbon of the 4-mehtylhexen-2-enyl residue [23]. The presence of this side chain was also supported by the long-range couplings observed in the HMBC spectrum (Table 1 and Figure S5) [13] between the two olefinic carbons C-3′ and C-4′, which appeared in the ^13^C NMR spectrum (Table 1 and Figure S3) as a singlet and a doublet at δ 130.5 and 138.0, respectively. C-3′ coupled with H_3_-2′, the first methyl of the side chain, which resonated at δ 12.4,CH_3_/1.81,br s whereas C-4′, coupled with the same methyl and the other (H_3_-8′), which was observed at δ 19.7,CH_3_/0.98, dFurthermore, in the COSY spectrum, an allylic coupling (*J* = 1.2 Hz) was observed between H-4′ and H_3_-2′. This 4-mehtylhexen-2-enyl side chain was bonded to the secondary carbon (HC-5), which resonated at δ 80.3,CH/4.37s of a trihydrofuran ring. This was supported by the HMBC coupling observed between C-2′, C-3′, and C-4′ with H-5. The presence of a trisubstituted furan ring was suggested by the typical signal of the two sp^3^ quaternary and one sp^2^ tertiary carbons as C-6, C-6a, and C-3a, which appeared in the ^13^C NMR spectrum at δ 68.1, 94.8, and 157.0, respectively. In the HMBC spectrum, C-6 correlated with H-5 and the geminal methyl group H_3_-9′, which resonated at δ 18.5,CH_3_/1.38,s; C-6a coupled with H-5 and C-3a coupled with H_3_-9′. The trihydrofuran ring was side-fused to a furanone ring, being the head-bridge carbons C-3a and C-6a. Consequently, the ^13^C NMR spectrum showed two sp^2^ carbons at δ 166.5, typical of a carbonyl (C-2) of an α,β-unsaturated γ-lactone ring and the other (C-3) at δ 98.4, typical of a tertiary carbon of a tetrasubstituted double bond, which was oxygenated [22]. These findings were confirmed by the couplings observed in the HMBC spectrum between both C-2 and C-3, with H_3_-1′, which is the residual vinyl methyl group bonded to C-3, resonating at δ 7.2,CH_3_/1.90,s. The presence of pentasubstituted dihydrofuro[3,2-b]furan-2(5*H*)-one in **1** was supported by the couplings observed in the HMBC spectrum between C-3a and C-6 with H_3_-9′ [21,22]. The investigation of the HSQC (Table 1and Figure S4) [13] spectrum allowed us to assign the chemical shifts to the protonated residual carbons; thus, the chemical shift values were assigned to all the protons and corresponding carbons, as listed in Table 1. Therefore, **1** was formulated as 6,6a-dihydroxy-3,6-dimethyl-5-(4-methylhex-2-en-2-yl)-6,6a-dihydrofuro[3,2-b]furan-2(5*H*)-one.

**Figure 2:**
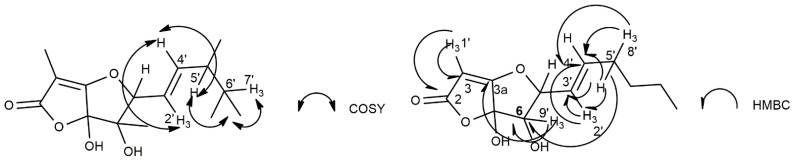
The most significant COSY and HMBC correlations observed in the corresponding spectra of **1**.

The structure assigned to araufuranone was supported by the other couplings observed in the HMBC spectrum (Table 1 and Figure 2) as well as from the data of its HR ESIMS spectrum (Figure S7). This latter spectrum showed the sodium adduct ion [M + Na]^+^ at 305.1370 *m*/*z*.

**Figure 3:**
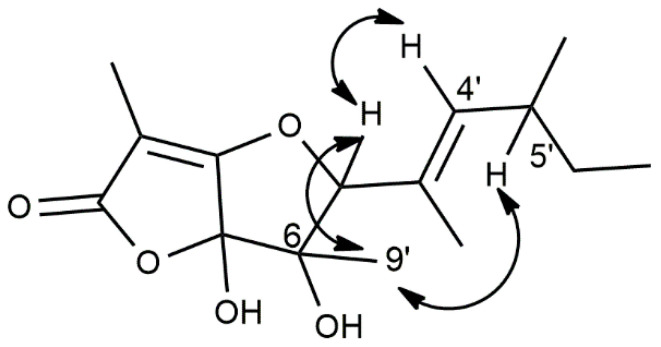
Significant NOE correlations observed in the NOESY spectrum of **1**.

The relative stereochemistry of **1** was determined by a NOESY spectrum (Figure 3 and Figure S6) [13]. As shown in Figure 3, the correlation between H-5 and H_3_-9′ allowed us to assign a *cis*-stereochemistry between the OH group at C-6 and the side chain bonded at C-5 of the trihydrofuran ring.

This was supported by the correlation observed between H-4′ and H-5. Furthermore, the already cited correlation between H-5 and H_3_-9′ and the lack of any correlation between H-4′ and H_3_-2′ allowed us to assign an *E*-stereochemistry to the double of the side chain. Considering the very low amount of **1**, which also withstood crystallisation and was used for the biological assays, it was not possible to assign its absolute configuration.

Fungal furans and furanones containing bioactive metabolites are well-known [24,25,26,27]; however, there are no natural products containing dihydrofuro[3,2-b]furan-2(5*H*)-one. Currently, there is only one deposited patent reporting the synthesis of dihydrofuranfuranone derivatives as antitumor agents [28]. Thus, araufuranone is the first fungal metabolite belonging to this group of natural compounds to be isolated for the first time from *A. araujiae*. The metabolites most similar to **1** are neovasinin and penpolonin analogues [29,30,31]

The two already known metabolites were identified as neovasinin and 2,4-dihydroxy-6-hydroxymethylbenzaldehyde (**2** and **3**, respectively; Figure 1) by a comparison of their physical and spectroscopic data with those reported in the literature by Nakajima et al. [29] and Ballantine et al. [32] for **2** and **3**, respectively. The attribution of the chemical shift of all the protons and corresponding carbon of **2** were supported by the couplings observed in its COSY, HSQC, and HMBC spectra [13]. In particular, its NOESY spectrum showed the correlation between Me-16 and H-10, which allowed us to confirm the *E*-configuration of the double bond of the side chain linked to C-7. Furthermore, the correlation observed between H-7 and Me-15, considering that the dihydropyran ring assumed a pseudochair conformation, allowed us to also confirm their axial–equatorial location or vice versa, and thus the relative configuration of R* and S* to C-6 and C-7, respectively. The ESIMS spectrum of neovasinin, recorded in a positive modality, showed the potassium [M + K]^+^, sodiated [M + Na]^+^, and protonated [M + H]^+^ adduct ions at *m*/*z* 347, 331, and 309, respectively. The same spectrum recorded in a negative modality showed the pseudomolecular ion [M − H]^−^ at *m*/*z* 307. The ESIMS spectrum of the trisubstituted benzaldehyde (**3**) showed the sodiated adduct ion [M + Na]^+^ at 191 *m*/*z*.

Neovasinin (**2**) was first isolated from *Neocosmospora vasinfecta* E. F. Smith, which is a fungal pathogen causing root- and fruit-rot as well as seedling damping-off in many crops (e.g., pepper, peanuts, and soybeans) [30]. From the same fungus was also isolated a previously undescribed α-pyrone, named neovasinone [31]. When assayed on lettuce (*Lactuca sativa)* seedlings, neovasinin weakly inhibited the root elongation [29] whereas neovasinone caused the stimulation of root seedlings [31]. These results demonstrate that the carbonyl group of the right α-pyrone ring present in neovasinone plays an important role in imparting activity [29]. The partial stereochemistry of neovasinin, which has been characterised by spectroscopic and chemical methods, was also reported from the same authors and a few of them also assigned its absolute configuration by a combination of an X-ray analysis and a degradation reaction [33]. Neovasinin was successively isolated, together with a plethora of other metabolites, from *Penicillium* sp. SYPF7381, which was obtained from the rhizosphere soil of *Pulsatilla chinensis* (Bunge) Regel collected from Huludao in the Liaoning province of China. Neovasinin showed a significant inhibition of NO production [34]. Similarly, **2** was also isolated, together with other metabolites from the co-culture of *Aspergillus fumigatus* D Fresenius and *Fusarium oxysporum* R1 Snyder & Hansen, collected from two traditional medicinal plants, *Edgeworthia chrysantha* Lindl. and *Rumex madaio* Makino. The metabolites did not show toxicity against human pathogens [35]. Finally, **2** was isolated, together with five undescribed α-pyrones named penpolonins A–E, from the endophytic fungus *Penicillium polonicum* Zalessky collected from the roots of *Camptotheca acuminata* Decne, showing a moderate cytotoxic activity [36]. Neovasinin showed phytotoxic activity [29]. Penpolonins were only assayed for cytotoxicity in comparison with neovasinin; their phytotoxic activity was not tested. Neovasinin and two penpolonins showed cytotoxic activity against the Hep-2 and TU212 human laryngeal cancer cell lines [36].

2,4-Dihydroxy-6-hydroxymethylbenzaldehyde (**3**) was previously isolated, together with a few other aromatic analogues, from the culture filtrates of *Aspergillus rugulosus* I.M.I. 84338 [21] and successively together with several other metabolites from *Clematis mandshurica* Rupr., which is a traditional crude drug for the treatment of urethritis, carbuncles, and carcinomas [37].

In the puncture assay at the tested concentrations (see above), araufuranone proved to be weakly active on the leaves of only two plant species, *Diplotaxis* sp. and *Sonchus* sp., whereas the other two metabolites were even less toxic and only on *Diplotaxis* (data not shown). On cress, compounds **2** and **3** were able to partially inhibit rootlet elongation (around 20%; data not shown) whereas araufuranone was almost inactive. None of the metabolites caused a reduction in the germination capability of the seeds. On *P. ramosa* seeds, the compounds were completely inactive at the tested concentrations. Although the phytotoxic activity of the three metabolites seemed to be plant species-dependent, the high activity of the fungal culture filtrates and corresponding organic extract (that could be due to additives or the synergistic activity of the three metabolites) suggested a further in-depth investigation is required on its activity aimed at its potential application as bioherbicides. Further studies should be also carried out on the phytotoxic and other potential biological activities of the pure metabolites. Furthermore, the cytotoxic activity of neovasinin also suggests its potential application in medicine, as well as to test the related aurofuranone to investigate this type of activity.

## 4. Conclusions

A new phytotoxic tetrasubstituted dihydrofuro[3,2-b]furan-2(5*H*)-one was isolated together with neovasinin and a trisubstituted benzaldehyde from *A. araujiae*, proposed as a potential mycoherbicide for the biocontrol of the noxious weed *A. hortorum*. Aurofuranone is, to the best of our knowledge, the first example of a naturally occurring compound showing the described carbon skeleton. It showed to be weakly active on the leaves of two weed species, *Diplotaxis* sp. and *Sonchus* sp.; the other two metabolites were even less toxic. However, the weak toxicity of the pure metabolites compared with the stronger biological activity of the fungal culture filtrates and the corresponding organic extract was not surprising because synergistic effects of the pool of metabolites are possible. Considering the results shown, the organic extract of *A. araujiae* deserves further attention to evaluate its potential to control weeds.

## Figures and Tables

**Table 1:** ^1^H and ^13^C NMR data of araufuranone (**1**) recorded in CDCl_3_ ^a,b^.

Position	δC ^c^	δH (J in Hz)	HMBC
2	166.5 C	-	CH_3_-1′
3	98.4 C	-	CH_3_-1′
3a	157.0 C	-	CH_3_-9′
5	80.3 CH	4.37 s	CH_3_-9′, CH_3_-2′
6	68.1 C	-	H-5, CH_3_-9′
6a	94.8 C	-	H-5
1′	7.2 CH_3_	1.90 s	-
2′	12.4 CH_3_	1.81 br s	H-5, H-4′
3′	130.5 C	-	H-5, CH_3_-2′
4′	138.0 CH	5.22 dd (9.4, 1.2)	H-5, CH_3_-2′, CH_3_-8′
5′	33.5 CH	2.43 m	CH_3_-7′, CH_3_-8′
6′	29.9 CH_2_	1.29 m	CH_3_-7′, CH_3_-8′
7′	11.3 CH_3_	0.91 t (7.3)	H-4′
8′	19.7 CH_3_	0.98 d (6.9)	H_2_C-6′
9′	18.5 CH_3_	1.38 s	H-5

^a^ The chemical shifts are in δ values (ppm) from TMS. ^b^ The 2D ^1^H-^1^H (COSY) and ^13^C-^1^H (HSQC) NMR experiments delineated by the correlations of all the protons and the corresponding carbons. ^c^ Multiplicities were assigned by DEPT spectrum.

## Supplementary Materials

The following supporting information can be downloaded at: https://www.mdpi.com/2218-273X/12/9/1274/s1, Figure S1. ^1^H NMR spectrum of araufuranone recorded in CD_3_OD at 400 MHz; Figure S2. COSY spectrum of araufuranone recorded in CD_3_OD at 400 MHz; Figure S3. ^13^C NMR spectrum of araufuranone recorded in CD_3_OD at 100 MHz; Figure S4. HSQC spectrum of araufuranone recorded in CD_3_OD at 400/100 MHz; Figure S5. HMBC spectrum of araufuranone recorded in CD_3_OD at 400/100 MHz; Figure S6. NOESY spectrum of araufuranone recorded in CD_3_OD at 400 MHz; Figure S7. HR ESIMS spectrum of araufuranone, recorded in positive modality.

## References

[1] Csurhes S., Edwards R. (1998). Potential Environmental Weeds in Australia: Candidate Species for Preventative Control.

[2] Bayón N.D., Arambarri A.M. (1999). Anatomy andethnobotany of the medicinal species of the Pampean province: Asclepiadaceae. Acta Farm. Bonaer.

[3] Endress M.E., Bruyns P.V. (2000). A revised classification of the Apocynaceae sl. Bot. Rev..

[4] Elorza M.S., Sánchez E.D.D., Sobrino Vesperinas E. (2004). Atlas de las Plantas Alóctonas Invasoras en España.

[5] Bucciarelli A., Cambi V.N., Villamil C.B. (2008). Morphoanatomy of *Araujia hortorum* E. Fourn. (Asclepiadaceae), native species with medicinal interest. Phyton Buenos Aires.

[6] Champion P.D., James T.K., Dawson M.I. (2010). New names for New Zealand weeds. N. Z. Plant Prot..

[7] Coombs G., Peter C.I. (2010). The invasive ‘mothcatcher’ (*Araujia sericifera* Brot.; Asclepiadoideae) co-opts native honeybees as its primary pollinator in South Africa. AoB Plants.

[8] Winks C.J., Fowler S.V. (2000). Prospects for Biological Control of Moth Plant, Araujia sericifera (Asclepiadaceae).

[9] Waipara N.W., Winks C.J., Gianotti A.F., Villamil C.B., Villamil S.C., Delhey R., Kiehr M., Traversa M.G., Carpintero D.L. (2006). Surveys for potential biocontrol agents for moth plant in New Zealand and Argentina. N. Z. Plant Prot..

[10] Anderson F.E., Santos López S.P., Sánchez R.M., Reinoso Fuentealba C.G., Barton J. (2016). *Puccinia araujiae*, a promising classical biocontrol agent for moth plant in New Zealand: Biology, host range and hyperparasitism by *Cladosporium uredinicola*. Biol. Control.

[11] Ramírez G.H., Anderson F.E. (2019). Characterization of an Ascochyta disease of the invasive vine *Araujia hortorum* E. Fourn. (Apocynaceae). J. King Saud Univ. Sci..

[12] Ramírez G.H., María Virginia Bianchinotti V., Anderson F.E. (2022). Single and combined effect of two fungal diseases on growth of moth plant, *Araujia hortorum* (Apocynaceae). N. Z. J. Bot..

[13] Berger S., Braun S. (2004). 200 and More Basic NMR Experiments: A Practical Course.

[14] Spegazzini C.L. (1882). Fungi argentini additis nonnullis brasiliensibus montevideensibusque. Pugillus Quartus An. Soc. Cient. Argent..

[15] de Hoog G.S., Gerrits van den Ende A.H.G. (1998). Molecular diagnostics of clinical strains of filamentous Basidiomycetes. Mycoses.

[16] White T.J., Bruns T., Lee S., Taylor J., Innis M.A., Gelfand D.H., Sninsky J.J., White T.J. (1990). Amplification and direct sequencing of fungal ribosomal RNA genes for phylogenetics. PCR Protocols: A Guide to Methods and Amplifications.

[17] Rehner S.A., Samuels G.J. (1994). Taxonomy and phylogeny of *Gliocladium* analysed from nuclear large subunit ribosomal DNA sequences. Mycol. Res..

[18] Vilgalys R., Hester M. (1990). Rapid genetic identification and mapping of enzymatically amplified ribosomal DNA from several *Cryptococcus* species. J. Bacteriol..

[19] Pinkerton F., Strobel G.A. (1976). Serinol as an activator of toxin production in attenuated cultures of *Heliminthosporium sacchari*. Proc. Natl. Acad. Sci. USA.

[20] Vurro M., Boari A., Pilgeram A.L., Sands D.C. (2006). Exogenous amino acids inhibit seed germination and tubercle formation by *Orobanche ramosa* (broomrape): Potential application for management of parasitic weeds. Biol. Control.

[21] Pretsch E., Buhlmann P., Affolter C. (2000). Structure Determination of Organic Compounds Tables of Spectral Data.

[22] Breitmaier E., Voelter W. (1987). Carbon-13 NMR Spectroscopy.

[23] Nakanishi K., Solomon P.H. (1977). Infrared Absorption Spectroscopy.

[24] Turner W.B., Aldridge D.C. (1983). Fungal Metabolite II.

[25] Dewick P.M. (2006). Medicinal Natural Products: A Biosynthetic Approach.

[26] Evidente A., Kornienko A., Lefranc F., Cimmino A., Dasari R., Evidente M., Mathieu V., Kiss R. (2015). Sesterterpenoids with anticancer activity. Curr. Med. Chem..

[27] Masi M., Maddau L., Linaldeddu B.T., Scanu B., Evidente A., Cimmino A. (2018). Bioactive metabolites from pathogenic and endophytic fungi of forest trees. Curr. Med. Chem..

[28] Gabriele B., Chimento A., Mancuso R., Pezzi V., Ziccarelli I., Sirianni R. (2019). Preparation of Dihydrofurofuranone Derivatives for Use as Antitumor Agents.

[29] Nakajima H., Nishimura K., Hamasaki T., Kimura Y., Udagawa S. (1987). Structure of neovasinin a new metabolite produced by the fungus, *Neocosmospora vasinfecta* E.F. Smith, and its biological activity to lettuce seedlings. Agric. Biol. Chem..

[30] Domsch K.H., Grams W., Anderson T.H. (1980). Compendium of Soil Fungi.

[31] Nakajima H., Nishimura K., Hamasaki T., Kimura Y., Yokota T., Udagawa S.I. (1987). Structure of neovasinone, a new α-pyrone plant growth regulator produced by the fungus, *Neocosmospora vasinfecta* EF Smith. Agric. Biol. Chem..

[32] Ballantine J.A., Hassall C.H., Jones B.D. (1968). The biosynthesis of phenols—XVII: Some phenolic metabolites of mutant strains of *Aspergillus regulosus*. Phytochemistry.

[33] Nakajima H., Fujuyama K., Kimura Y., Hamasaki T. (1992). Absolute stereochemistry of neovasinin, a phytotoxin produced by the fungus, Neocosmospora vasinfecta. Biosci. Biotech. Biochem..

[34] Feng Q.M., Li X.Y., Li B.X., Zhang T.Y., Wang H.F., Zhang M.Y., Wu Y.Y., Chen G., Zhang Y.X., Pei Y.H. (2020). Isolation and identification of two new compounds from the *Penicillium* sp. SYPF7381. Nat. Prod. Res..

[35] Yu R., Li M., Wang Y., Bai X., Chen J., Li X., Wang H., Zhang H. (2021). Chemical investigation of a co-culture of *Aspergillus fumigatus* D and *Fusarium oxysporum* R1. Rec. Nat. Prod..

[36] Ma Y., Wen Y., Cheng H., Deng J., Peng Y., Bahetejiang Y., Huang H., Wu C., Yang X., Pang K. (2021). Penpolonins A–E, cytotoxic α-pyrone derivatives from *Penicillium polonicum*. Bioorg. Med. Chem. Lett..

[37] Weon J.B., Jung Y.S., Ma C.J. (2016). Neuroprotective activity of compounds of *Clematis mandshurica* against glutamate-induced cell death in HT22 cells. Planta Med..

