# Correction: Masi et al. Araufuranone: A New Phytotoxic Tetrasubstituted Dihydrofuro[3,2-b]furan-2(*5H*)-One Isolated from *Ascochyta araujiae*. *Biomolecules* 2022, *12*, 1274

**DOI:** 10.3390/biom13050844

**Published:** 2023-05-16

**Authors:** Marco Masi, Angela Boari, Francisco Sautua, Marcelo Anibal Carmona, Maurizio Vurro, Antonio Evidente

**Affiliations:** 1 Department of Chemical Sciences, University of Naples Federico II, Complesso Universitario Monte Sant’Angelo, Via Cintia 4, 80126 Napoli, Italy; 2 Institute of Sciences of Food Production, National Research Council, Via Amendola, 122/O, 70126 Bari, Italy; 3 Phytopathology, University of Buenos Aires, Buenos Aires C1053, Argentina

In the original article [1], there was a mistake in Figure 1 as published. Both the incorrect and the correct Figure 1 were printed. The corrected Figure 1 appears below. 

The authors state that the scientific conclusions are unaffected. This correction was approved by the Academic Editor. The original publication has also been updated.

## Figures and Tables

**Figure 1:**
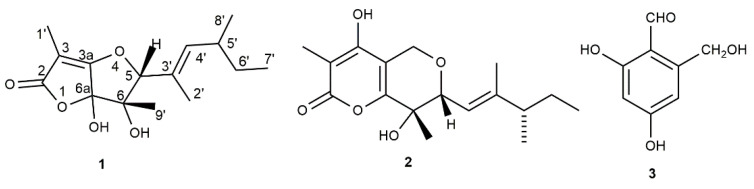
Structures of araufuranone (**1**), neovasinin (**2**), and 2,4-dihydroxy-6-hydroxymethylbenzaldehyde (**3**).
