# Supplementary material for: Araufuranone: A New Phytotoxic Tetrasubstituted Dihydrofuro[3,2-b]furan-2(5H)-One Isolated from Ascochyta araujiae
Source: Biomolecules. 2022 Sep 10;12(9):1274. doi: 10.3390/biom12091274 (PMC9496196; doi:10.3390/biom12091274)
Supplement: Supplementary file 1 [file biomolecules-12-01274-s001.zip › biomolecules-1837773-supplementary.pdf]

Supporting Information

Araufuranone: A New Phytotoxic Tetrasubstituted Dihydrofuro[3,2-b]furan-2(5*H*)-One Isolated from *Ascochyta araujiae*

Marco Masi [1], Angela Boari [2], Francisco Sautua [3], Marcelo Anibal Carmona [3], Maurizio Vurro [2] and Antonio Evidente [1,*]

Index

Figure S1. ^{1}H NMR spectrum of araufuranone recorded in CD_3OD at 400 MHz.

Figure S2. COSY spectrum of araufuranone recorded in CD_3OD at 400 MHz.

Figure S3. ^{13}C NMR spectrum of araufuranone recorded in CD_3OD at 100 MHz.

Figure S4. HSQC spectrum of araufuranone recorded in CD_3OD at 400/100 MHz.

Figure S5. HMBC spectrum of araufuranone recorded in CD_3OD at 400/100 MHz.

Figure S6. NOESY spectrum of araufuranone recorded in CD_3OD at 400 MHz.

Figure S7. HR ESIMS spectrum of araufuranone, recorded in positive modality.

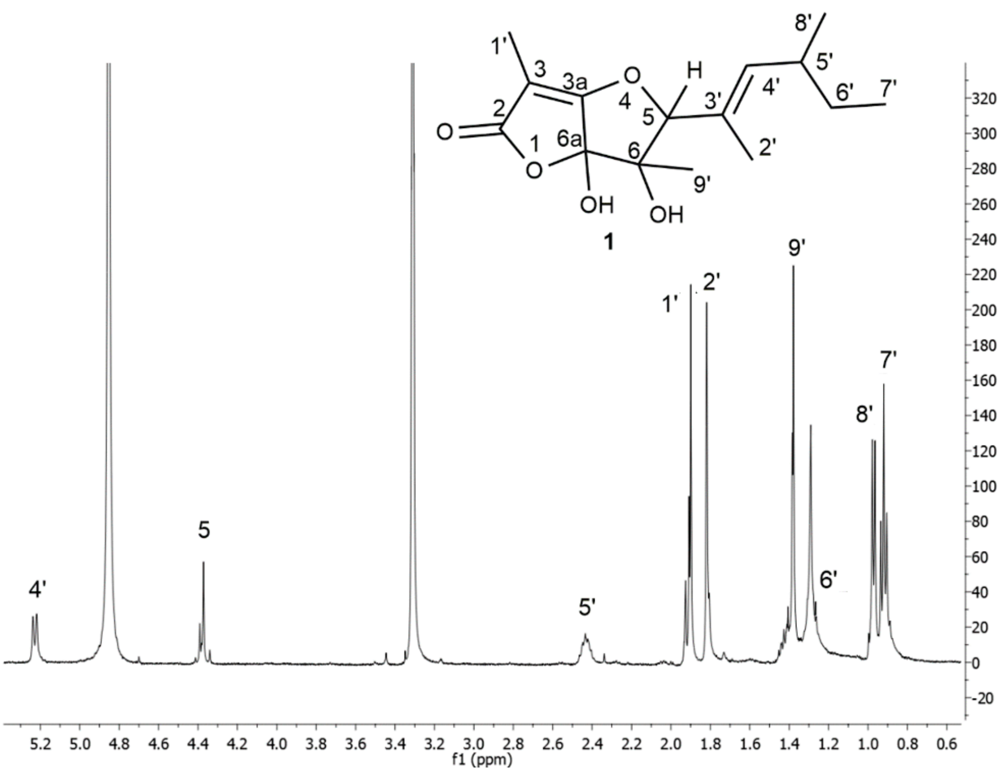

Figure S1. ^{1}H NMR spectrum of araufuranone recorded in CD_3OD at 400 MHz.

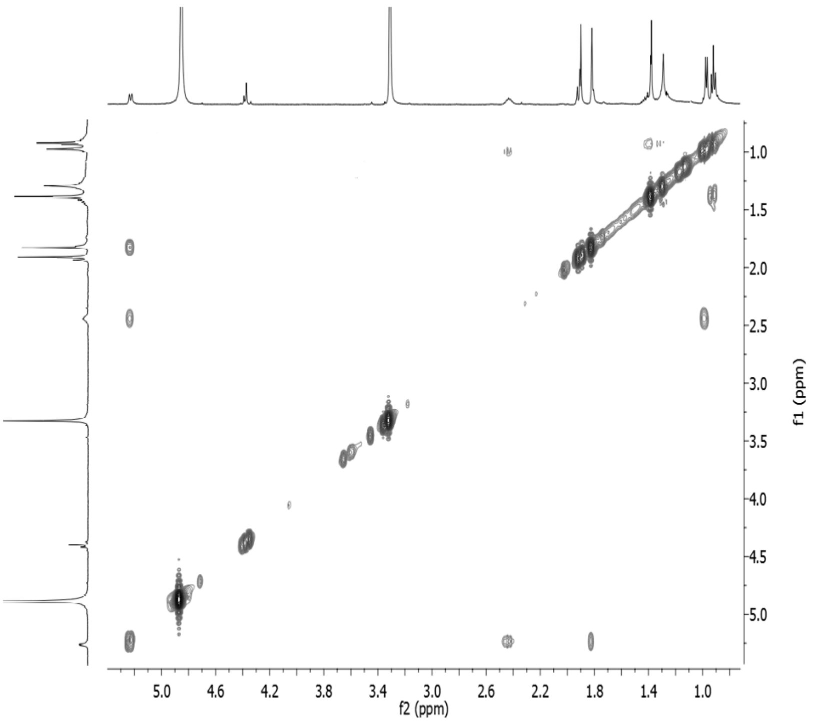

Figure S2. COSY spectrum of araufuranone recorded in CD_3OD at 400 MHz.

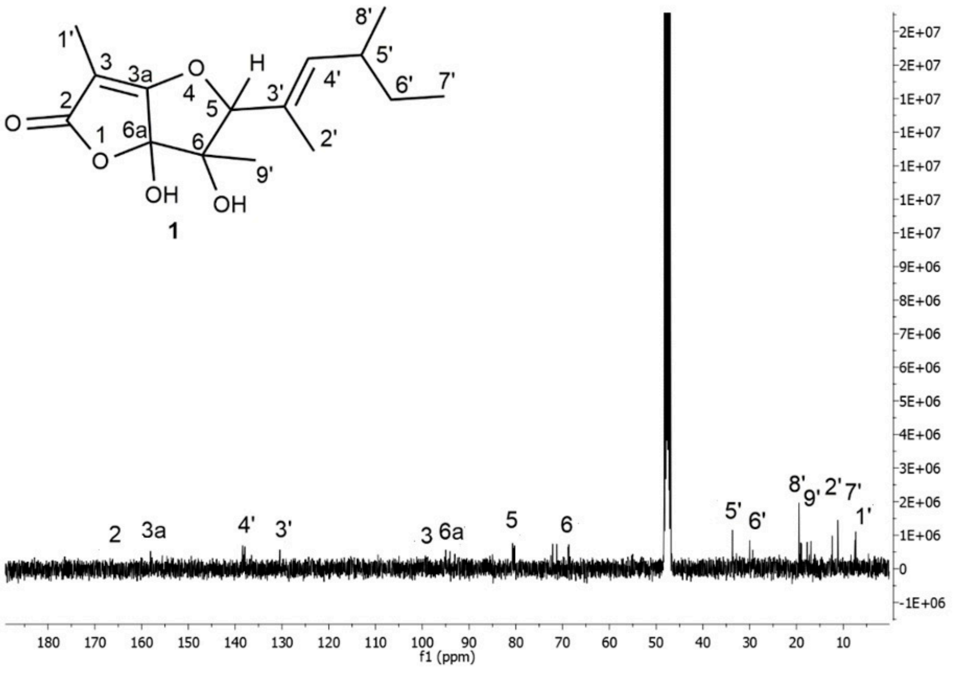

Figure S3. ^{13}C NMR spectrum of araufuranone recorded in CD_3OD at 100 MHz.

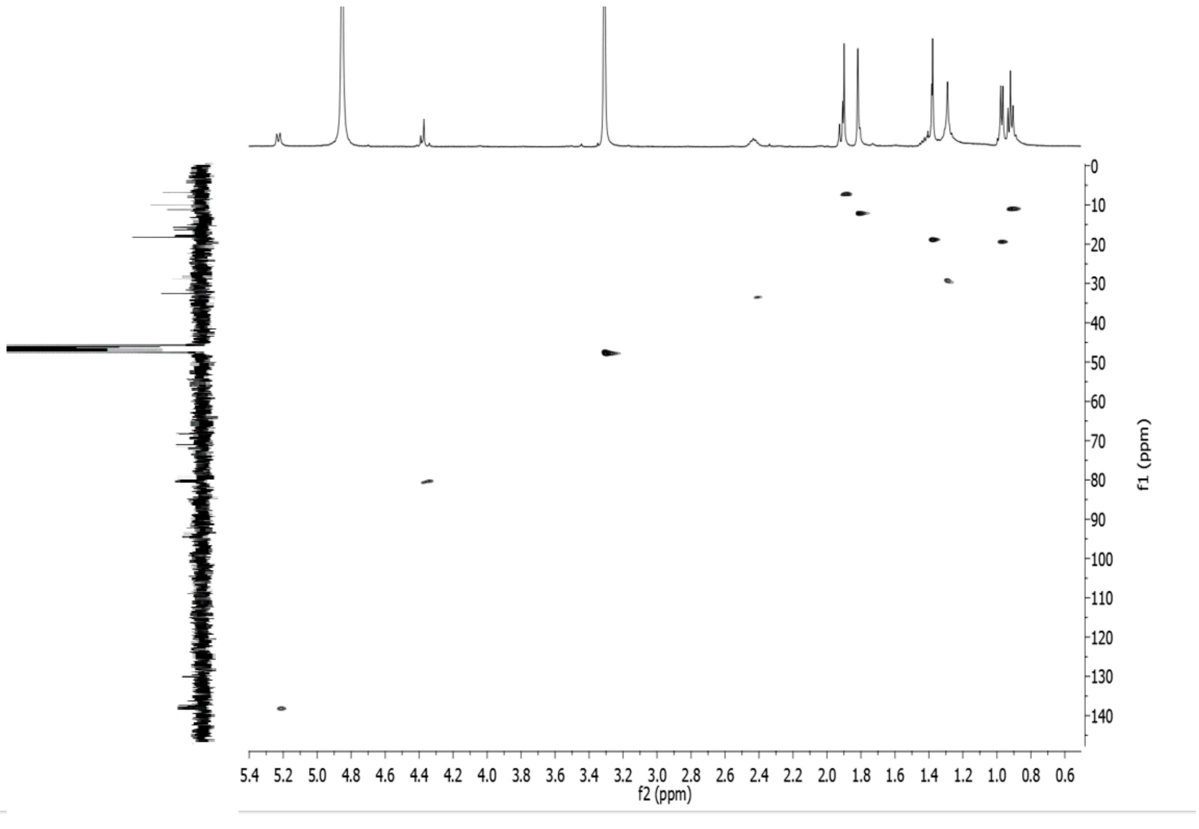

Figure S4. HSQC spectrum of araufuranone recorded in CD_3OD at 400/100 MHz.

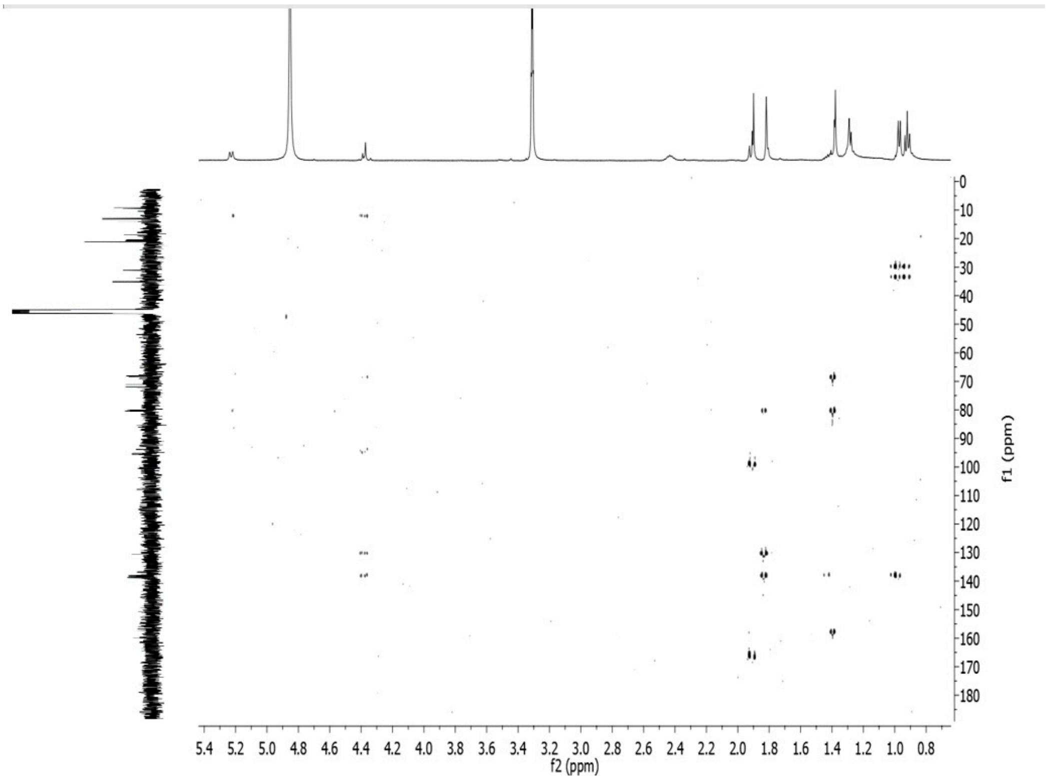

Figure S5. HMBC spectrum of araufuranone recorded in CD_3OD at 400/100 MHz.

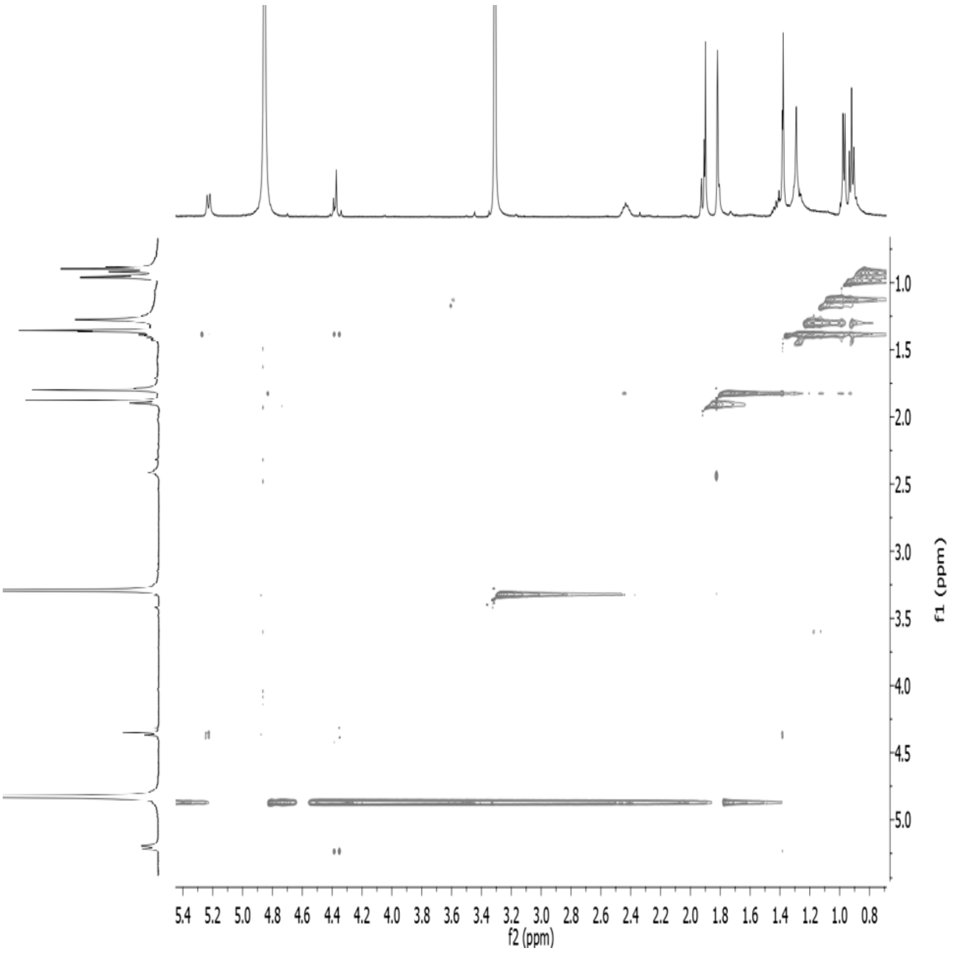

Figure S6. NOESY spectrum of araufuranone recorded in CD_3OD at 400 MHz.

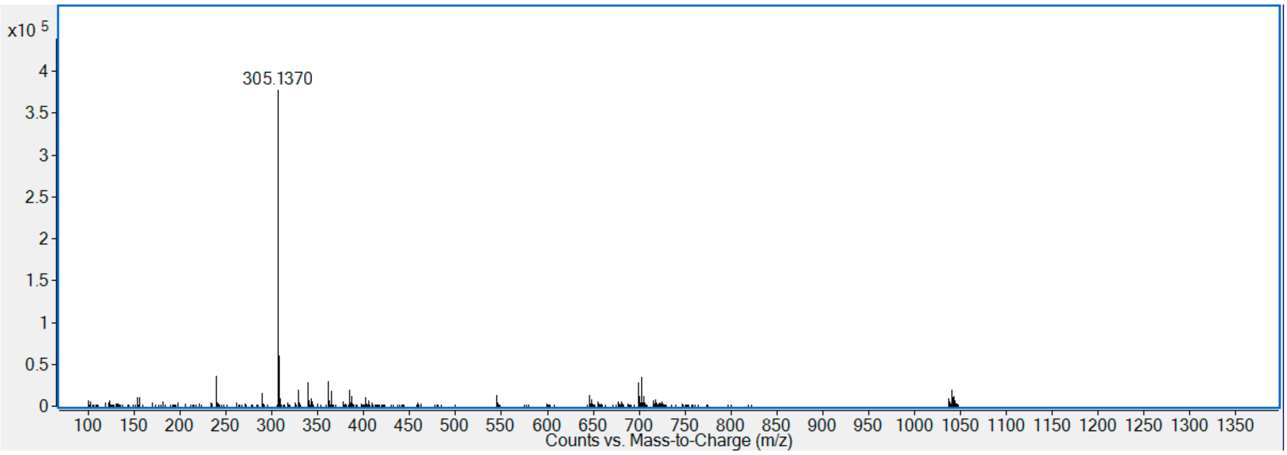

Figure S7. HR ESIMS spectrum of araufuranone, recorded in positive modality.
